# Hypoxia-Induced Pulmonary Injury—Adrenergic Blockade Attenuates Nitrosative Stress, and Proinflammatory Cytokines but Not Pulmonary Edema

**DOI:** 10.3390/jcdd11070195

**Published:** 2024-06-27

**Authors:** Isabel Riha, Aida Salameh, Annekathrin Hoschke, Coralie Raffort, Julia Koedel, Beate Rassler

**Affiliations:** 1 Carl-Ludwig-Institute of Physiology, University of Leipzig, 04103 Leipzig, Germany; isabel.riha@yahoo.de (I.R.); anne.hoschke@web.de (A.H.); 2 Department of Pediatric Cardiology, Heart Centre, University of Leipzig, 04289 Leipzig, Germany; aida.salameh@medizin.uni-leipzig.de (A.S.); coralie.raffort@uni-leipzig.de (C.R.); 3 Institute of Pathology, University of Leipzig, 04103 Leipzig, Germany; julia.koedel@medizin.uni-leipzig.de

**Keywords:** normobaric hypoxia, adrenergic blockade, pulmonary edema, pulmonary inflammation, tumor necrosis factor α, nitrotyrosine, left and right ventricular catheterization

## Abstract

Hypoxia can induce pulmonary edema (PE) and inflammation. Furthermore, hypoxia depresses left ventricular (LV) inotropy despite sympathetic activation. To study the role of hypoxic sympathetic activation, we investigated the effects of hypoxia with and without adrenergic blockade (AB) on cardiovascular dysfunction and lung injury, i.e., pulmonary edema, congestion, inflammation, and nitrosative stress. Eighty-six female rats were exposed for 72 h to normoxia or normobaric hypoxia and received infusions with NaCl, prazosin, propranolol, or prazosin–propranolol combination. We evaluated hemodynamic function and performed histological and immunohistochemical analyses of the lung. Hypoxia significantly depressed LV but not right ventricular (RV) inotropic and lusitropic functions. AB significantly decreased LV function in both normoxia and hypoxia. AB effects on RV were weaker. Hypoxic rats showed signs of moderate PE and inflammation. This was accompanied by elevated levels of tumor necrosis factor α (TNFα) and nitrotyrosine, a marker of nitrosative stress in the lungs. In hypoxia, all types of AB markedly reduced both TNFα and nitrotyrosine. However, AB did not attenuate PE. The results suggest that hypoxia-induced sympathetic activation contributes to inflammation and nitrosative stress in the lungs but not to PE. We suggest that AB in hypoxia aggravates hypoxia-induced inotropic LV dysfunction and backlog into the pulmonary circulation, thus promoting PE.

## 1. Introduction

Acute exposure to hypoxia can induce pulmonary edema (PE) as can often be observed after rapid ascents to high altitude. High-altitude pulmonary edema (HAPE) is considered to be non-cardiogenic edema caused by elevated pulmonary capillary pressure resulting from hypoxic pulmonary vasoconstriction (HPV) [1,2]. HPV mainly occurs in pulmonary resistance arterioles but has also been observed in venules [3,4,5]. While pulmonary areas with high HPV have reduced capillary perfusion, relative overperfusion and increased capillary pressure occur in lung areas with mild HPV. The pathogenesis of HAPE is related to an uneven distribution of HPV [6]. If venous resistance in these areas is elevated, water accumulation in the lung and fluid filtration into the pulmonary interstitium may ensue [7]. Chest radiographs or computerized tomography scans in HAPE patients show a patchy distribution of edema, which is in accordance with the assumption of regionally heterogeneous pulmonary perfusion in hypoxia [1]. Moreover, subjects who are prone to develop HAPE, often show enhanced HPV accompanied by an excessive rise in pulmonary artery systolic pressure, which may aggravate fluid extravasation [8,9]. In less severe cases, HAPE is confined to the pulmonary interstitium. However, an excessive increase in capillary pressure may cause ultrastructural damage to the alveolo-capillary walls resulting in a high-permeability form of edema—a mechanism referred to as capillary stress failure [10,11]. The increase in pulmonary capillary pressure and hence, the formation of PE can be promoted by cardiac dysfunction such as an imbalance in the pumping function of the left and right ventricles as demonstrated in previous studies [12,13].

The precise mechanisms of HPV are not completely understood. Reduced bioavailability of nitric oxide (NO) is considered to be an important component of vasoconstriction [14,15,16]. In addition to its vasodilator properties, NO is also a potent scavenger of oxidizing radicals generated by peroxynitrite [17]. Low NO levels have been suggested to result from continual consumption by interaction between NO and the superoxide anion resulting in increased peroxynitrite formation [18,19]. Peroxynitrite radicals can induce DNA damage and apoptosis and are indicated by elevated levels of nitrotyrosine, an important marker of nitrosative stress [20]. Numerous studies on animals and humans indicated a close relationship between the generation of reactive oxygen and nitrogen species and hypoxia-induced pulmonary inflammation and edema [21,22,23,24,25]. 

Hypoxia-induced PE is typically accompanied by inflammatory reactions in the lungs [26,27]. This was confirmed in previous studies on rats in normobaric hypoxia. PE emerged after 6 h of hypoxia and deteriorated with prolonged exposure to hypoxia over 24 h [12]. It was accompanied by inflammation, as demonstrated by increased expression of pro-inflammatory cytokines such as interleukin (IL)-6 and tumor necrosis factor (TNF) α [13,28]. Inflammation, however, is considered to be a consequence rather than a cause of PE, but it may maintain and aggravate edema formation [29].

In addition, hypoxia induces the activation of the sympathetic nervous system [30,31,32]. Increased sympathetic activation, mainly mediated via α-adrenergic efferent pathways, is considered to play an important role in pulmonary vasoconstriction [33]. However, strong sympathetic activation may induce PE even in normoxic conditions. In human pathology, neurogenic PE and PE as a complication of pheochromocytoma are well-known examples of catecholamine-induced PE [34,35,36,37]. In rats, infusion of adrenergic agonists, in particular, α-adrenergic agonists, induced PE, inflammation, and later, fibrosis and vascular hypertrophy in the lungs [38,39]. We therefore hypothesized that adrenergic blockade (AB) might attenuate hypoxia-induced pulmonary injury including both edema and inflammation and that these beneficial effects would be strongest with α-AB.

While our previous studies [12,13] were designed to investigate cardiopulmonary reactions to hypoxia in an early stage of hypoxia exposure (up to 24 h), the present study focused on the following period of time (i.e., up to three days). The main objective of this study was to identify the contribution of hypoxic sympathetic activation to cardiopulmonary dysfunction under hypoxic conditions. The first question was whether hypoxia-induced pulmonary edema and inflammation are still present after 72 h of normobaric hypoxia. Secondly, we compared the effects of AB in normoxia and hypoxia on pulmonary edema and inflammation. In addition, we investigated whether nitrosative stress as indicated by increased levels of nitrotyrosine (NT) is involved in hypoxia-induced pulmonary injury. We also examined hemodynamic function for hypoxia-induced disturbances in order to assess their role in the formation of hypoxic PE. 

## 2. Materials and Methods

### 2.1. Animal Model

All experiments were performed on 86 female Sprague–Dawley rats supplied by Charles River (Sulzfeld, Germany). The body weight was 236 ± 1.3 g at the beginning of this study, corresponding to an age of about 10–12 weeks. All animal protocols were approved by the Federal State Agency (Landesdirektion Sachsen, protocol number TVV 46/18). The experiments were conducted in accordance with the Guide for the Care and Use of Laboratory Animals published by the National Institutes of Health and with the “European Convention for the Protection of Vertebrate Animals used for Experimental and other Scientific Purposes” (Council of Europe No 123, Strasbourg 1985).

### 2.2. Study Protocol

**Figure 1:**
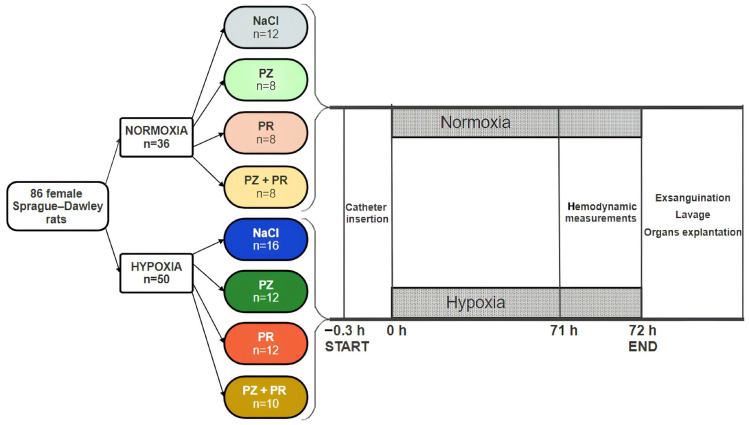
Study flow chart. The cohorts and the groups within each cohort (normoxia and hypoxia) are given along with the number of animals (n) per cohort/group. PZ prazosin, PR propranolol.

Animals were subdivided into two cohorts for exposure to normoxia (N) or normobaric hypoxia (H) for 72 h. For exposure to hypoxia, the animals were placed into a hypoxic chamber sized 65 × 105 × 50 cm. The gas mixture in the chamber contained 10% oxygen in nitrogen. Special equipment prevented penetration of ambient air during manipulations on the animals to keep the oxygen concentration in the chamber stable at 10 ± 0.5%. Normoxic animals remained under room air conditions. Additionally, all animals received an intravenous infusion over the total experimental time. The normoxic and hypoxic cohorts were subdivided into 4 groups each. From both cohorts, one group was infused with 0.9% sodium chloride (NaCl) solution (N-NaCl, H-NaCl). The remaining groups (one group each from the normoxic and hypoxic cohorts) were infused with the α-adrenergic blocker prazosin (PZ, 0.1 mg kg^−1^ h^−1^; N-PZ, H-PZ), the β-adrenergic blocker propranolol (PR, 0.16 mg kg^−1^ h^−1^; N-PR, H-PR), or a combination of the two (PZ+PR, 0.1 + 0.16 mg kg^−1^ h^−1^, respectively; N-PZ+PR, H-PZ+PR, see Figure 1).

The infusions were administered with automatic pumps (Infors AG, Basel, Switzerland) at a rate of 0.1 mL h^−1^ via an infusion catheter (Vygon, Aachen, Germany). The infusion catheter was inserted into the left jugular vein. This operation was performed with 2% isofluran anesthesia. After catheter insertion, the animals woke up and moved freely with access to tap water and a rat chow diet (Altromin C100, Altromin GmbH, Lage, Germany). Exposure to a hypoxic environment started immediately after catheter insertion.

### 2.3. Hemodynamic Measurements

About 50 min before the end of the exposure time, the animals were anesthetized with an intraperitoneal injection of thiopental (Trapanal^®^ 80 mg kg^−1^). They were tracheotomized, and a polyethylene cannula was placed in the trachea. The right ventricle (RV) was catheterized with a Millar^®^ (Millar Instruments, Houston, TX, USA) ultraminiature catheter pressure transducer (for more details see our previous publication [40]). For catheterization of the left ventricle (LV), we used a pressure-volume catheter (Millar Instruments, Houston, TX, USA). Data acquisition and analysis were performed with Power Lab and Lab Chart Software from ADInstruments (version 8.1.9, ADInstruments Europe/UK, Oxford, UK) and a modified LabChart Software (version 1.0) from the ADInstruments sales department (FMI Föhr Medical Instruments GmbH, Seeheim, Germany). Parallel conductance was corrected by injection of isotonic saline solution (0.1 mL, 0.9% NaCl). In both ventricles, we determined systolic peak pressures in the left and right ventricles (LVSP, RVSP), maximal velocities of increase (dP/dtmax), and decrease in pressure (dP/dtmin) as measures of ventricular contractility and relaxation, respectively, and heart rate (HR). In addition, end-diastolic pressure (LV edP) and stroke volume (SV) were measured in the LV. SV was calibrated by the thermodilution method (for more details, see [41]). After the withdrawal of the LV catheter tip into the aorta, diastolic aortic pressure (DAP) was measured to calculate mean aortic pressure (MAP). The cardiac index (CI, body mass-related cardiac output) was determined by thermodilution using a thermosensitive 1.5F microprobe and a Cardiomax II computer (Columbus Instruments, Columbus, OH, USA). Total peripheral resistance (TPR) was calculated by dividing MAP by the CI. Hypoxic animals remained in hypoxia until the completion of hemodynamic measurements.

### 2.4. Sampling of Materials

After completion of the hemodynamic measurements, the abdominal cavity was opened by midline incision. Animals were sacrificed by drawing blood from the abdominal aorta. Then, we opened the thoracic wall and collected pleural fluid. After ligation of the right main bronchus, a bronchoalveolar lavage (BAL) of the left lung was performed two times consecutively with 3 mL 0.9% NaCl each. The fluid was instilled via the tracheal cannula into the left lung and withdrawn immediately. The recovery rate was about 90% on average. The recovered BAL fluid was frozen and stored at −80 °C for BAL cytology and other analyses. The left lung was discarded thereafter. From the intact right lung, tissue samples were fixated in formalin for histological analysis. The histological examination served to detect blood congestion, inflammation, and edema in the lung. A piece of the middle lobe was taken for the determination of the wet-to-dry weight (W/D) ratio. 

### 2.5. Lung Histology

The formalin-fixated tissue samples of the right lung were embedded in paraffin, sliced, and stained with hematoxylin–eosin. Histological assessments were performed by two independent investigators (I.R. and J.K.) who were blinded to the treatment group. In the lungs, they evaluated PE and congestion. For a detailed quantification of PE, the complete histological section of a lung was assessed. First, PE severity (expressed as PE score) in each area of the section was gauged visually by evaluating the width of the alveolar septa and the definition of alveolar spaces. PE scores ranged from 0 (absent) to 1 (mild: alveolar septa slightly thickened, alveolar space well defined), 2 (moderate: thickness of alveolar septa about double the normal width, alveolar space narrowed but still defined), and 3 (severe: alveolar spaces hardly determinable and/or alveolar edema). The PE index (PEI) was calculated by cumulating the products of the PE score and the proportionate area of each part of the histological preparation. The congestion index (ConI) was determined in an analogous way. The results are based on the evaluation of all animals (numbers are stated in Figure 1).

### 2.6. Immunohistochemistry

The expressions of TNFα, a potent pro-inflammatory cytokine, and NT in the lungs were determined using immunohistochemistry. Specimens from the right lung, fixed with 4% formalin, were embedded in paraffin, and 2 μm slices were cut. After mounting on microscopic slides, the samples were dewaxed and rehydrated. For the determination of TNFα, the specimens were cooked in 0.01 M citrate buffer (pH = 6) and then blocked with bovine serum albumin (BSA) to saturate unspecific bindings. Antigen retrieval was performed according to [42]. The specimens were treated with rabbit monoclonal anti-TNFα primary antibody (1:100, Sigma-Aldrich, Taufkirchen, Germany) overnight at 4 °C. Next, the specimens were washed again in phosphate buffer and the appropriate goat anti-rabbit secondary antibody (1:200, Sigma-Aldrich, Taufkirchen, Germany) labeled with horseradish peroxidase (HRP) was applied for 2 h. After a further washing step, a peroxidase reaction was carried out using the red chromogen 3-amino-9-ethylcarbazole (AEC, Dako, Hamburg, Germany) according to the manufacturer’s instructions. Cell nuclei were counterstained with hemalum.

For the detection of NT, the dewaxed and rehydrated specimens were cooked in 0.01 M citrate buffer (pH = 6). After blockade with BSA to saturate unspecific bindings, the specimens were treated with mouse monoclonal anti-nitrotyrosine primary antibody (1:100, Merck-Millipore, Darmstadt, Germany) overnight at 4 °C. Thereafter, the specimens were washed, and the appropriate HRP-labeled secondary goat anti-mouse antibody (1:200, Sigma-Aldrich, Taufkirchen, Germany) was applied for 2 h at room temperature. Subsequently, the specimens were washed again, and the visualization of positive cells was performed with AEC red chromogen (Dako, Hamburg, Germany). Cell nuclei were counterstained with hemalum. The specimens were investigated microscopically using the Axioimager M1 microscope from Zeiss (Carl Zeiss, Jena, Germany). For the determination of TNFα, which is mainly located in the bronchial and peribronchial regions, photographs were taken from these regions using an AxioCam MRc 5 camera and AxioVision Rel. 4.6 software (Carl Zeiss, Jena, Germany) at 5× magnification. At least 10 pictures per animal were evaluated by a blinded observer (I.R.). For measurements in the pictures, the program ImageJ [43] was used. As TNFα is located within the cytosol, we determined the TNFα-positive area (given in μm^2^). The expression of TNFα is given as the TNFα-positive area related to the total lung area of the specimen (in percent). For NT determination, the procedure was similar, but photographs were taken at 20× magnification. From each animal, at least 50 photographs were evaluated to measure the total area of the specimen and the percentage of the NT-positive area. The results are based on the evaluation of all animals (numbers are stated in Figure 1).

### 2.7. Lung Wet-to-Dry Weight Ratio

Lung tissue samples were weighed immediately after preparation (wet weight, W) and after drying in an oven at 75 °C for 48 h (dry weight, D). The W/D ratio served as a surrogate parameter of water accumulation in the lung and, thus, as a second indicator of pulmonary edema in addition to histological edema markers.

### 2.8. BAL Cytology

BAL fluid was centrifuged for 20 min at 1500 rpm in a cytocentrifuge. The cytologic preparation was stained with hematoxylin–eosin and evaluated by a pathologist (J.K.) who was not aware of the treatment of the animal. The number of macrophages, neutrophils, lymphocytes, and eosinophils was given in percent of the total cell number.

### 2.9. Statistical Analysis

All data are presented as mean ± SEM. Statistical analyses were carried out with the software package SigmaPlot Version 14.0 (Systat Software GmbH, Erkrath, Germany) for Windows. All groups were statistically compared using analysis of variance (ANOVA) procedures. At first, we performed a Shapiro–Wilk test of normality. If the data were normally distributed, we used a one-way ANOVA with a post hoc test according to Fisher’s method of least significant differences (LSD). If the data were not normally distributed, a Kruskal–Wallis ANOVA on ranks with a post hoc test according to Dunn’s method was applied. Both post hoc tests are multiple comparison procedures comparing all possible pairwise mean differences. *p* values < 0.05 were considered significant. For comparisons between the normoxic and hypoxic cohorts, we used Student’s t-test if the data were normally distributed and Mann–Whitney’s U-test if they were not normally distributed.

## 3. Results

### 3.1. Hemodynamic Results

All hemodynamic results are presented in Table 1. In general, cardiac function, particularly LV function, was compromised under hypoxic conditions. Hypoxia significantly depressed the inotropic and lusitropic functions of the LV. In animals without AB, LVSP decreased from 123 ± 3 mm Hg in normoxia to 103 ± 4 mm Hg in hypoxia (*p* = 0.005). Significant reductions were also found for LV dP/dt max, as a measure of contractility, and for stroke volume. MAP was also lower in hypoxic than in normoxic control rats. The HR significantly decreased from 442 ± 8 min^−1^ in the N-NaCl group to 410 ± 8 min^−1^ in the H-NaCl group (*p* = 0.028). Consequently, the CI was also significantly reduced in the hypoxic control group (313 ± 16 mL min^−1^ kg^−1^ as compared with 387 ± 12 mL min^−1^ kg^−1^ in the N-NaCl group). LV dP/dt min, as an indicator of relaxation, was mildly decreased while LV edP was slightly elevated. In contrast, RV function was not compromised by hypoxia; on the contrary, RVSP was even slightly improved.

AB decreased LV function significantly even in normoxia. This effect was strongest with β-AB (alone or in combination with the α-blocker PZ). The effects on the RV were weaker and mostly not significant. In hypoxia, LVSP was not further reduced by AB, but LV dP/dt max and LV dP/dt min decreased even more than without AB, in particular, with β-AB. Similarly, the stroke volume, HR, and CI decreased further with AB compared with the hypoxic control group. TPR tended to increase under hypoxic conditions, above all with β-AB (Table 1). 

### 3.2. Lung Histology and W/D Ratio

**Figure 2:**
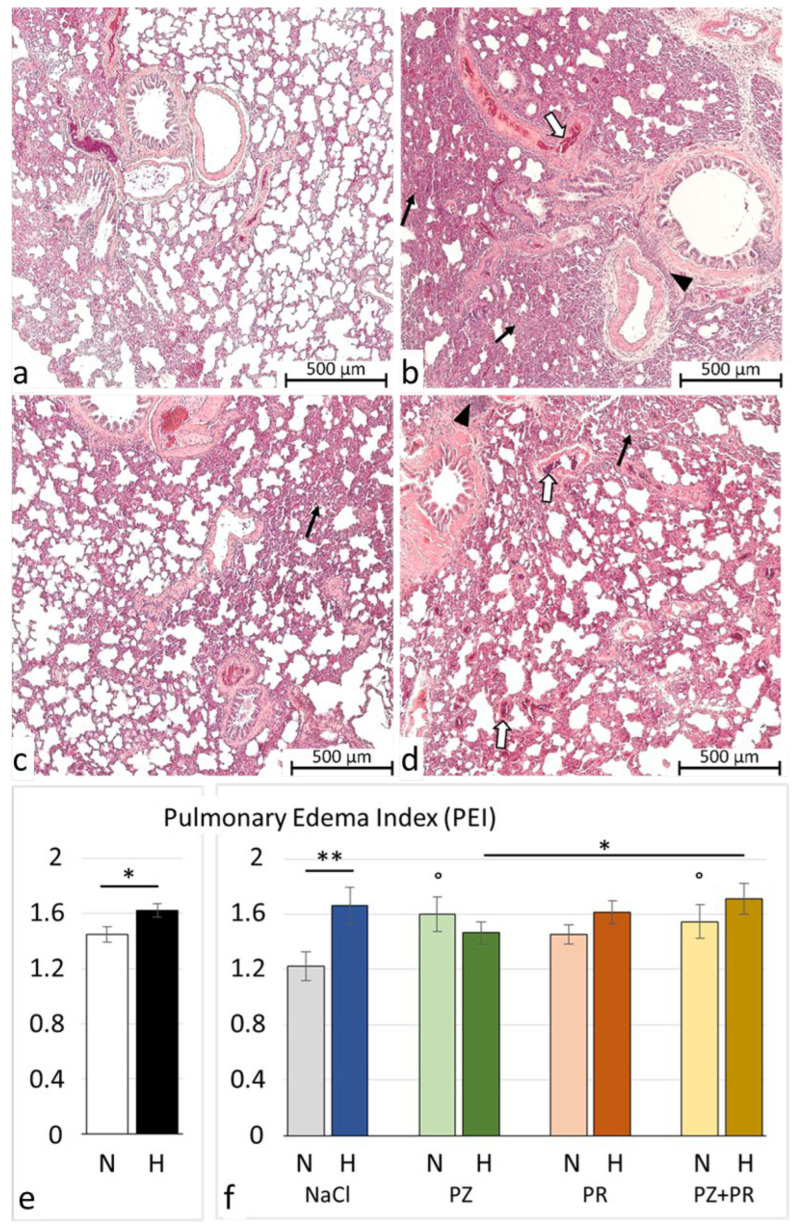
Pulmonary injury. (**a**–**d**) Representative histological lung images (5 * magnification): (**a**) N-NaCl: largely unaffected lung with only mild edema and congestion; (**b**) H-NaCl: moderate to severe edema and inflammation, moderate congestion; (**c**) N-PZ+PR: mild to moderate edema and congestion, mild inflammation; and (**d**) H-PZ+PR: moderate to severe edema and congestion, moderate inflammation. Pulmonary edema is marked by black arrows; inflammation is marked by white arrows; and blood congestion is marked by arrowheads. (**e**,**f**) Pulmonary edema index of cohorts (**e**) and groups (**f**) (mean ± SEM): N normoxia, H hypoxia, PZ prazosin, PR propranolol. Statistics: comparison of cohorts: t-test; comparison of groups: one-way ANOVA with post hoc Fisher’s LSD test. Significance marks: ― significant difference between marked columns, * *p* < 0.05; ** *p* < 0.01; ° significant difference compared with the corresponding control (NaCl) group, *p* < 0.05.

**Figure 3:**
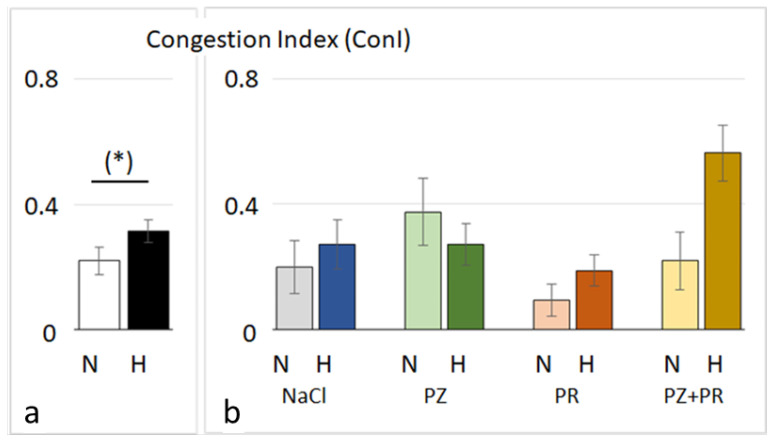
Lung congestion index of cohorts (**a**) and groups (**b**) (mean ± SEM): N normoxia, H hypoxia, PZ prazosin, PR propranolol. Statistics: comparison of cohorts: Mann–Whitney rank sum test; comparison of groups: Kruskal–Wallis ANOVA on ranks with post hoc Dunn’s test. Significance marks: (*) the difference between the normoxic and hypoxic cohorts is near significance (*p* = 0.05).

After 72 h of normobaric hypoxia, histology of the lungs showed mild to moderate PE associated with signs of inflammation. The edema was largely confined to the interstitium, while alveoli were scarcely affected. Noteworthily, even the lungs of the normoxic animals were not free from edema, but the edema was significantly more severe in the hypoxic cohort (*p* = 0.023, Figure 2). The most pronounced difference between the normoxic and hypoxic groups was observed in the control groups (N-NaCl vs. H-NaCl: *p* = 0.003). Application of adrenergic blockers aggravated the edema in the normoxic animals; this was even significant with PZ (*p* = 0.017) and PZ+PR (*p* = 0.041). In contrast to what we had expected, AB did not attenuate PE under hypoxic conditions. We found a slight reduction in PE in the H-PZ group. By contrast, PE was even slightly aggravated with PZ+PR and was significantly more severe in the H-PZ+PR group than in the H-PZ group (*p* = 0.026, Figure 2). The edema was associated with congestion of blood. Congestion was more severe in the hypoxic cohort than in the normoxic cohort (*p* = 0.05) and was most pronounced in the H-PZ+PR group (Figure 3). The W/D ratio of the lungs corresponded with the histological findings. In very rare cases, the W/D ratio was greater than 6, which indicates severe alveolar edema. However, the W/D ratio was significantly lower in the normoxic cohort than in the hypoxic cohort (Table 2). Correspondingly, the amount of pleural fluid was slightly higher in most of the normoxic animals than in the hypoxic animals, in particular, with AB, indicating that fluid drainage from the lungs was more efficient under normoxia compared with hypoxic conditions (Table 2). BAL cytology showed unremarkable results characterized by a vast majority of macrophages (about 90%); the rest was attributable to lymphocytes, neutrophil granulocytes, and a very small portion of eosinophil and basophil granulocytes. The cell distribution was similar in all animal groups and corresponded to normal BAL cytology findings in humans [44].

### 3.3. Immunohistochemistry

**Figure 4:**
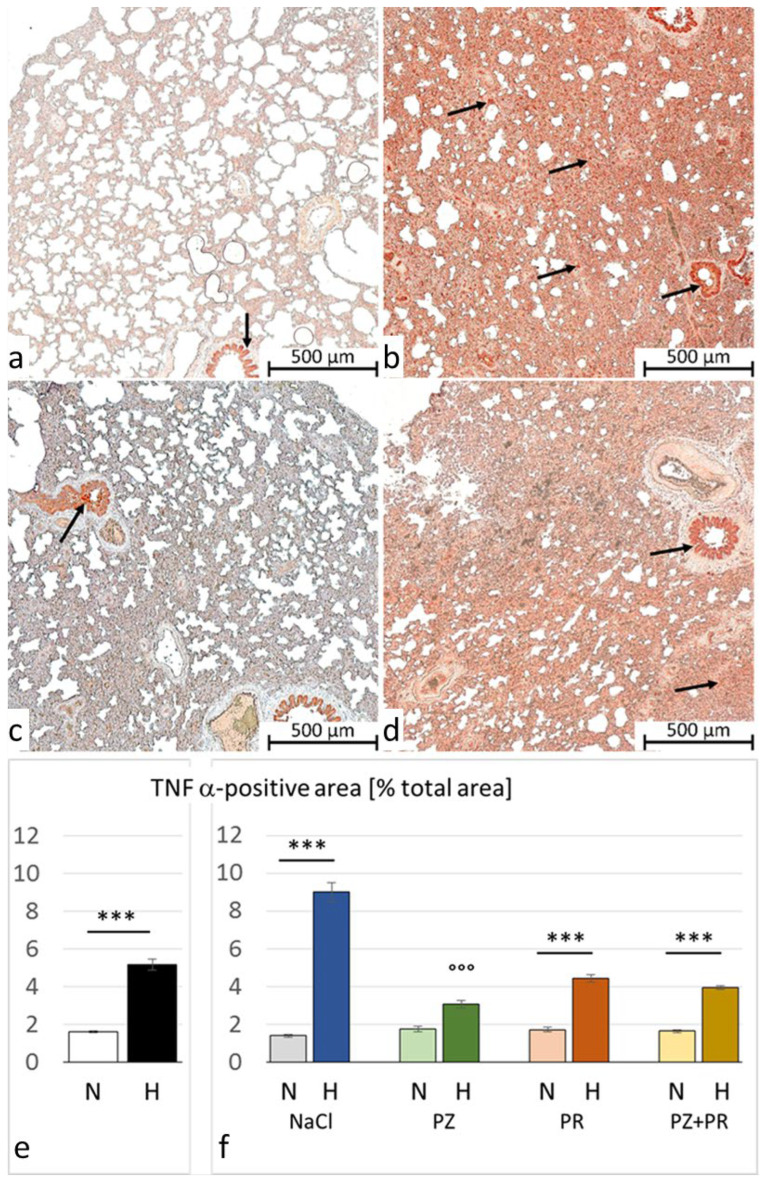
Tumor necrosis factor (TNF) α in the lung. (**a**–**d**) Representative immunohistochemical images from (**a**) N-NaCl; (**b**) H-NaCl; (**c**) N-PZ+PR; and (**d**) H-PZ+PR; all slices are at 5× magnification; red staining indicates TNFα-positive cells (examples are marked by arrows). (**e**,**f**) The abundance of TNFα of cohorts (**e**) and groups (**f**) expressed as a percentage of positive area related to the total lung area (mean ± SEM): N normoxia, H hypoxia, PZ prazosin, PR propranolol. Statistics: comparison of cohorts: Mann–Whitney rank sum test; comparison of groups: Kruskal–Wallis ANOVA on ranks with post hoc Dunn’s test. Significance marks: ― significant difference between marked columns, *** *p* < 0.001; °°° significant difference compared with the corresponding control (NaCl) group, *p* < 0.001.

Immunohistochemical analysis, however, revealed a significant increase in TNFα expression in the lungs of hypoxic rats, indicating the existence of inflammation as can be seen in the histological images (see Figure 2). The results showed a more than threefold percentage of TNFα-positive lung areas in the hypoxic cohort compared to the normoxic one. This difference was most pronounced in the control groups with a more than five-fold positive area in the H-NaCl group compared with the N-NaCl animals (*p* < 0.001). AB had no effect on the normoxic animals but attenuated TNFα expression in the hypoxic groups. This effect was particularly strong with single α-AB (H-PZ: *p* < 0.001 compared with H-NaCl; Figure 4).

**Figure 5:**
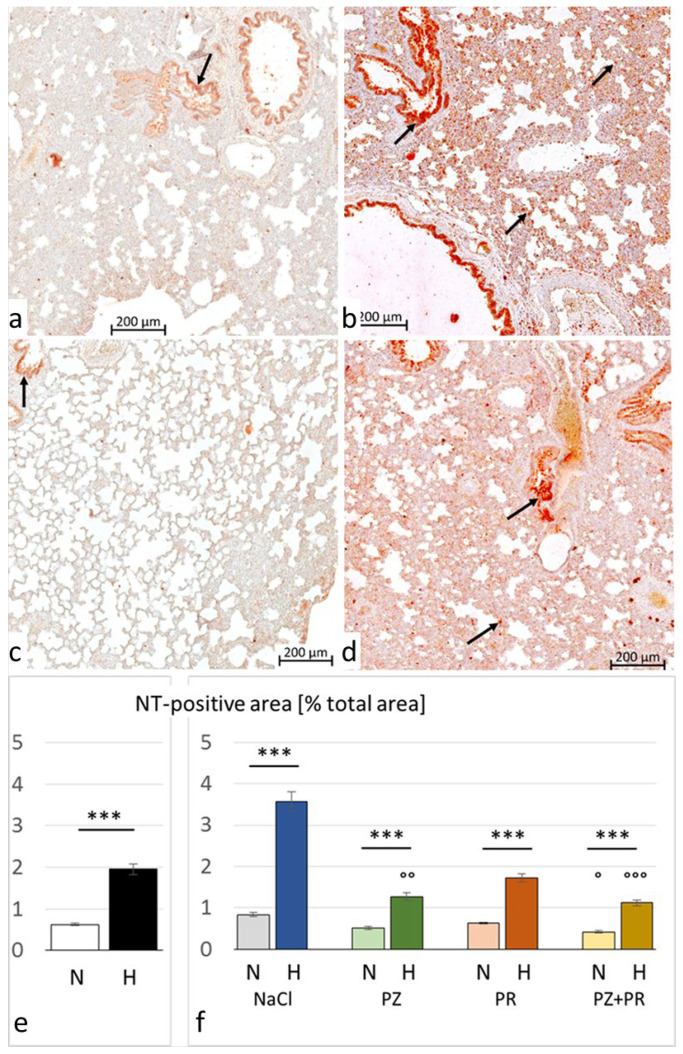
Nitrotyrosine (NT) in the lung. (**a**–**d**) Representative immunohistochemical images from (**a**) N-NaCl; (**b**) H-NaCl; (**c**) N-PZ+PR; and (**d**) H-PZ+PR; all slices are at 20× magnification; red staining indicates NT-positive cells (examples are marked by arrows). (**e**,**f**) The abundance of NT of cohorts (**e**) and groups (**f**) expressed as a percentage of positive area related to the total lung area (mean ± SEM): N normoxia, H hypoxia, PZ prazosin, PR propranolol. Statistics: comparison of cohorts: Mann–Whitney rank sum test; comparison of groups: Kruskal–Wallis ANOVA on ranks with post hoc Dunn’s test. Significance marks: ― significant difference between marked columns, *** *p* < 0.001; ° significant difference compared with the corresponding control (NaCl) group, *p* < 0.05, °° *p* < 0.01, °°° *p* < 0.001.

Hypoxia-induced nitrosative stress in the lungs is indicated by a significant increase in NT. In the hypoxic cohort, the percentage of NT-positive cells was more than three-fold the percentage in normoxic animals. In the H-NaCl group, NT-positive cells were more than four times the value in the N-NaCl group. Similar to TNFα, AB attenuated nitrosative stress under hypoxia. A significant reduction in the percentage of NT-positive cells as compared with the H-NaCl group was achieved with PZ (*p* = 0.002) and PZ+PR (*p* < 0.001). Of note, PZ+PR reduced NT even in normoxic conditions (*p* = 0.043; Figure 5).

## 4. Discussion

Our results showed that edema in the rat lung was still present after 72 h of exposure to normobaric hypoxia. PE was assessed by two independent methods including the W/D ratio and lung histology. Histology indicated predominantly interstitial and almost no alveolar PE. This was confirmed by the W/D ratio, which was about 5 in the hypoxic cohort (see Table 2). The discriminatory power of the W/D ratio does not differentiate well between normal lungs and interstitial edema but between interstitial and alveolar edema. A W/D ratio of 6 or higher certainly indicates a severe alveolar PE [45], which was only very rarely observed here. 

The severity of PE was in a similar range or even slightly greater than observed in a previous study after 24 h of hypoxia [12]. This indicates that the processes involved in edema formation are still active after 72 h of hypoxia and promote the progression of pulmonary injury. Uneven HPV associated with elevated pulmonary capillary pressure is considered to be the main cause of hypoxic PE such as HAPE [1,6]. Of note, a specific effect of barometric pressure within the body is highly improbable, and the main causative factor for hypoxia-induced PE is not a reduction in barometric pressure but a reduction in inspiratory pO_2_ [46], suggesting that the pathogenesis of PE is comparable in normobaric and hypobaric hypoxia. Pulmonary vasoconstriction induces blood congestion in the pulmonary circulation, as was also demonstrated in the hypoxic animals of the present study. Elevated end-diastolic pressure in the LV of hypoxic rats confirmed our assumption of a backlog from the LV into the pulmonary circulation. Moreover, hypoxia affected the hemodynamics of the LV and RV in a differential manner, thus inducing a mismatch in the inotropic functions of the LV and RV that aggravated pulmonary congestion. While LV systolic pressure and contractility were significantly reduced under hypoxic conditions, RV systolic pressure and contractility were maintained or even slightly improved. It should be mentioned here that the hemodynamic function of the normoxic control group (N-NaCl) was in line with values reported by our group and others as typical for young SD rats [12,38,47,48,49].

Noteworthily, in contrast to our hypothesis, PE was not significantly attenuated by AB. And more, AB even aggravated PE under normoxic conditions. The hemodynamic results showed that AB, in particular, with β-blockers, also compromised cardiac inotropic and lusitropic functions in normoxia. In general, the LV was more severely affected than the RV. As to be expected, β-AB (alone or in combination with the α-blocker PZ) exerted the most pronounced effects, particularly on LV contractility and relaxation. Isolated α-AB had weaker effects on LV function but reduced TPR, indicating a vasodilatory effect. Vasodilation in the pulmonary vasculature in combination with a reduced LV relaxation and slightly elevated LV edP suggests increased congestion in the pulmonary vascular bed [28]. These alterations of cardiovascular functions by adrenergic blockers and the resulting backlog into the lungs may explain the aggravation of PE.

In hypoxia, adrenergic blockers further deteriorated hypoxia-induced LV depression. Again, the depression was more pronounced with β-adrenergic blockers and predominantly concerned the LV. As a consequence, stroke volume decreased and LV edP increased compared with the hypoxic animals with maintained adrenergic effects. The highest LV edP values were found in the H-PZ+PR group, and the congestion index also reached its highest values in this group. These results confirm our suggestion that AB has reinforced the backlog into the pulmonary circulation. This effect is even stronger in hypoxia than in normoxia and prevents the regression of edema.

Hypoxic PE is accompanied by inflammation in the lungs [26,27,50]. This was also confirmed in our experiments. Lung histology showed the presence of inflammation, which was underpinned by significantly elevated levels of TNFα in lung tissue. TNFα is a potent stimulator of inflammatory responses in many pulmonary diseases such as asthma, chronic obstructive pulmonary disease (COPD), and acute lung injury/acute respiratory distress syndrome (ALI/ARDS) but also HAPE and high-altitude pulmonary hypertension [51,52]. It is widely accepted that hypoxia-induced PE is not induced by inflammatory processes but is maintained and aggravated by inflammation [29]. Inflammation begins at an early stage of hypoxia exposure almost at the same time as PE. This was previously shown in rats exposed to normobaric hypoxia with an oxygen content of 10% over time intervals between 6 and 24 h by the parallel occurrence of PE and histological signs of inflammation as well as by increased expression of TNFα [13,28]. The present results show that inflammatory processes are still active after 72 h of hypoxia and can maintain edema formation. Animal studies with chronic hypoxia demonstrated the persistence of pulmonary inflammation. The serum levels of TNFα were increased after two weeks of hypoxia [53]. With longer hypoxia exposure, the expression of proinflammatory cytokines in the rat lung further increased, forming a chronic inflammatory microenvironment in pulmonary arteries, which promoted the accumulation of fibrosis-associated molecules [54]. These processes finally result in vascular cell hypertrophy, pulmonary vascular remodeling, and fibrosis as well as pulmonary hypertension [55]. 

Activation of the sympathetic nervous system under hypoxic conditions can make an important contribution to hypoxia-induced pulmonary inflammation. Catecholamines are potent inflammatory activators of nuclear factor-κB (NF-κB) in macrophages, thus increasing the release of proinflammatory cytokines such as TNFα [56]. The proinflammatory effects of catecholamines or sympathetic activation are predominantly induced by α-adrenergic stimulation, while stimulation of β-adrenoceptors mainly exerts anti-inflammatory effects [38,57]. However, β-adrenergic stimulation can also induce inflammation under certain conditions [58,59]. Previous studies on rats demonstrated that experimental infusion of NE and other adrenergic agonists induced pulmonary inflammation and the upregulation of proinflammatory cytokines in serum and lung tissue [60,61]. Accordingly, our results demonstrated that sympathetic blockade, particularly α-AB with prazosin, significantly reduced TNFα levels in the lungs of hypoxic rats (see Figure 4).

Besides pulmonary edema and inflammation, hypoxia increases the generation of free radicals, thus inducing oxidative/nitrosative stress. A study on rats revealed a significant increase in vascular leakage, in proinflammatory cytokines such as IL-6 and TNFα, in the expression of NF-κB, and in reactive oxygen species in the lungs of rats exposed for up to 24 h to hypobaric hypoxia at 280 mmHg [22]. Moreover, animal studies demonstrated that hypoxia induced NO-synthase (NOS) isoforms in various organs, resulting in an increased production of peroxynitrite [20,62,63]. Peroxynitrite radicals induce nitrosylation of tyrosine residues, thus generating nitrotyrosines, which further can induce cell death.

Enhanced nitrosative stress, increased levels of TNFα, and sympathetic stimulation are closely interrelated. TNFα is known to induce free radical generation and induction of oxidative/nitrosative stress [51,64], as demonstrated in various tissues including the lung [65,66,67]. From human pathology, such as Takotsubo syndrome, it is known that an excessive release of catecholamines can induce both inflammation and nitrosative stress [68]. Rat studies demonstrated that the administration of adrenergic agonists increased both inflammation and nitrosative stress, while AB attenuated it [69,70]. This is in accordance with our results demonstrating that AB reduced in parallel both TNFα and NT levels in pulmonary tissue. The results clearly show that hypoxic sympathetic activation makes a considerable contribution to hypoxia-induced lung injury.

There are some limitations of this study. Firstly, we only used female rats. This was performed to compare the results with previous studies [12,13,28,38,39,40]. Previous rat studies have demonstrated that the organs and functions investigated in the present study did not significantly differ between male and female animals. Under hypoxic conditions, blood pressure and heart rate were similar in male and female rats [71]. An analysis of 142 heart, lung, vascular, kidney, and blood phenotypes revealed similar variability in these traits between male and female rats [72]. Secondly, flushing of the lungs before extraction is recommended to remove blood remnants from the lungs, which may appear as artifacts in lung histology. However, it was important for us to prove blood congestion in the lungs of hypoxic rats. We are aware that without flushing, some blood residues can also remain in the lungs of normoxic controls, which can be misinterpreted as true congestion. In the hypoxic groups, however, congestion is real and would probably be removed by flushing. The comparison between normoxic controls and treatment groups allows a semi-quantitative estimation of the degree of congestion in hypoxic animals. Further, we intended to determine the lung wet-to-dry weight (W/D) ratio as a second indicator of pulmonary edema. Flushing of the lungs might falsify lung wet weight. For these two reasons, we refrained from flushing the lungs in all animals. Finally, the reduction in the proinflammatory cytokine TNFα under AB treatment suggests attenuation of pulmonary inflammation. However, the sole evaluation of TNFα does not allow us to draw reliable conclusions about the development of inflammation. Future analyses should include further markers of inflammation such as IL-6 and NF-κB.

## 5. Conclusions

The present results show that pulmonary edema and inflammation are still persistent after 72 h of normobaric hypoxia. This injury is associated with enhanced nitrosative stress, as indicated by increased levels of nitrotyrosine in the lungs. AB, in particular, α-AB, reduced both the expression of TNFα and NT in the lungs. However, these advantageous effects were not accompanied by attenuation of PE. This finding is in accordance with the pathogenetic concept of HAPE, as this type of edema is formed independently of inflammation. The failure of AB to reduce pulmonary edema might be explained by the hemodynamic effects of AB aggravating the hypoxia-induced inotropic dysfunction of the LV and the backlog into the pulmonary circulation. These findings may have important clinical implications for people under sympatholytic medication, which may apply to travelers to high altitudes, and even more for patients suffering from hypoxia and hypoxemia.

## Figures and Tables

**Table 1:** Hemodynamic results.

Cohort	Normoxic Cohort:				Hypoxic Cohort:			
Group	N-NaCl	N-PZ	N-PR	N-PZ+PR	H-NaCl	H-PZ	H-PR	H-PZ+PR
LVSP [mmHg]	**106.9 ± 3.7**				**99.4 ± 2.4**			
	123.3 ± 3.4	99.9 ± 5.5 °°	102.2 ± 11.9 °	92.5 ± 5.2 °°°	103.5 ± 3.8 **	96.8 ± 4.4	95.5 ± 6.3	101.3 ± 5.5
LV dP/dtmax [mmHg/s]	**8748 ± 494**				**7142 ± 364** **			
	10,419 ± 622	9912 ± 933	6943 ± 1029 °°	6804 ± 908 °°	8365 ± 569 *	7798 ± 755	5838 ± 551 °°	5893 ± 858 °
LV dP/dtmin [mmHg/s]	**−9534 ± 541**				**−8437 ± 413**			
	−11,896 ± 468	−9328 ± 1170 °	−8433 ± 1307 °°	−7136 ± 927 °°°	−10,176 ± 614	−9178 ± 801	−6957 ± 689 °°	−6439 ± 788 °°°
Stroke volume [μL]	**194.7 ± 6.9**				**165.5 ± 6.5** **			
	215.3 ± 7.2	211.7 ± 6.8	167.4 ± 18.8 °	172.4 ± 13.2 °	185.6 ± 11.6 *	152.9 ± 8.8 ° **	143.5 ± 18.5 °	164.8 ± 12.3
LV edP [mmHg]	**5.74 ± 0.34**				**6.97 ± 0.25** ***			
	5.93 ± 0.54	6.50 ± 1.13	5.04 ± 0.54	5.52 ± 0.67	6.38 ± 0.35	7.06 ± 0.48	7.10 ± 0.34	7.69 ± 0.86
RVSP [mmHg]	**27.9 ± 0.7**				**30.8 ± 0.9** *			
	30.9 ± 0.9	26.8 ± 1.1	27.7 ± 1.6	24.7 ± 1.0 °	34.3 ± 1.5	29.6 ± 1.9	28.3 ± 0.8	29.1 ± 2.6
RV dP/dtmax [mmHg/s]	**2201 ± 138**				**2096 ± 119**			
	2307 ± 183	2466 ± 280	2542 ± 444	1511 ± 66	2458 ± 192	2350 ± 247	1638 ± 99	1646 ± 309 °
RV dP/dtmin [mmHg/s]	**−1777 ± 114**				**−1692 ± 81**			
	−2098 ± 174	−1815 ± 214	−1503 ± 129	−1503 ± 315	−1928 ± 137	−1824 ± 181	−1377 ± 80	−1469 ± 191
Heart rate [min^−1^]	**426.0 ± 7.7**				**390.0 ± 6.2** ***			
	441.8 ± 8.1	458.1 ± 11.2	423.9 ± 9.3	374.5 ± 21.1 °°°	410.4 ± 8.3 *	407.4 ± 11.1	359.6 ± 11.9 *** °°°	367.7 ± 13.9 °°
Cardiac index [ml min^−1^ kg^−1^]	**337.4 ± 14.5**				**264.8 ± 9.6** ***			
	387.4 ± 11.8	360.2 ± 38.6	302.3 ± 27.7 °°	276.5 ± 25.9 °°°	312.7 ± 15.6 **	258.7 ± 12.9 *** °	243.4 ± 18.0 °°	245.0 ± 14.7 °°
MAP [mmHg]	**96.4 ± 3.5**				**91.0 ± 2.4**			
	109.6 ± 3.5	91.0 ± 5.7	92.7 ± 11.6	84.7 ± 5.0	91.4 ± 3.6	91.2 ± 4.8	87.1 ± 5.9	95.2 ± 5.7
TPR [mmHg·min·kg·s^−1^]	**0.30 ± 0.01**				**0.38 ± 0.03** *			
	0.29 ± 0.01	0.26 ± 0.02	0.33 ± 0.07	0.31 ± 0.03	0.30 ± 0.02	0.35 ± 0.03	0.42 ± 0.08	0.40 ± 0.04

Values are given as mean ± SEM. LV, RV: left and right ventricle, respectively. SP: systolic peak pressure; dP/dt max: maximal velocity of pressure increase; dP/dt min: maximal velocity of pressure decrease; edP: end-diastolic pressure; MAP: mean aortic pressure; TPR: total peripheral resistance. Cohort values are given in bold; group values are given in standard type. Significance marks: significant differences in hypoxic to the corresponding normoxic groups/cohorts: * *p* < 0.05; ** *p* < 0.01; *** *p* < 0.001; significant differences in blocker groups compared with the related (normoxic or hypoxic) control are marked with circles: ° *p* < 0.05; °° *p* < 0.01; °°° *p* < 0.001.

**Table 2:** Lung wet-to-dry weight (W/D) ratio and pleural fluid volume.

Cohort	Normoxic Cohort:				Hypoxic Cohort:			
Group	N-NaCl	N-PZ	N-PR	N-PZ+PR	H-NaCl	H-PZ	H-PR	H-PZ+PR
W/D ratio	**4.82 (4.73; 4.96)**				**5.03 (4.83; 5.42)** **			
	4.96 (4.80; 5.59)	4.77 (4.68; 4.82)	4.85 (4.66; 4.98)	4.79 (4.68; 4.92)	4.99 (4.82; 5.44)	5.01 (4.97; 5.16)	5.43 4.94; 5.74)	4.93 (4.73; 5.14)
Pleural fluid volume [mL]	**0.18 (0.10; 1.68)**				**0.20 (0.10; 1.23)**			
	0.10 (0.05; 0.16)	1.90 (0.13; 5.25)	0.35 (0.08; 1.68)	0.63 (0.16; 3.68)	0.13 (0.04; 0.55)	0.20 (0.08; 2.10)	0.20 (0.10; 0.80)	0.23 (0.10; 0.13)

Values are given as medians (25th/75th percentile). Cohort values are given in bold; group values are given in standard type. Significance marks: significant differences in the hypoxic cohort compared with the corresponding normoxic cohort: ** *p* < 0.01.

## Data Availability

The data are available on request from the corresponding author.

## Abbreviations

AB	adrenergic blockade
ALI/ARDS	acute lung injury/acute respiratory distress syndrome
BAL	bronchoalveolar lavage
BSA	bovine serum albumin
CI	cardiac index
ConI	congestion index
COPD	chronic obstructive pulmonary disease
DAP	diastolic aortic pressure
dP/dtmax	maximal velocity of increase in pressure
dP/dtmin	maximal velocity of decrease in pressure
edP	end-diastolic pressure
H	normobaric hypoxia
HAPE	high-altitude pulmonary edema
HPV	hypoxic pulmonary vasoconstriction
HR	heart rate
IL	interleukin
LV	left ventricle/ventricular
MAP	mean aortic pressure
N	normoxia
NaCl	sodium chloride
NE	norepinephrine
NF-κB	nuclear factor-κB
NO	nitric oxide
NOS	nitric oxide synthase
NT	nitrotyrosine
PE	pulmonary edema
PEI	pulmonary edema index
PR	propranolol
PZ	prazosin
RV	right ventricle/ventricular
SP	systolic peak pressure
SV	stroke volume
TNF	tumor necrosis factor
TPR	total peripheral resistance
W/D ratio	wet-to-dry weight ratio
